# Genomic structure and expression of uncoupling protein 2 genes in rainbow trout (*Oncorhynchus mykiss*)

**DOI:** 10.1186/1471-2164-7-203

**Published:** 2006-08-09

**Authors:** Issa Coulibaly, Scott A Gahr, Yniv Palti, Jianbo Yao, Caird E Rexroad

**Affiliations:** 1 West Virginia University, Animal and Veterinary Sciences Division, Po Box 6108, Morgantown, WV 26506, USA; 2 National Center for Cool and Cold Water Aquaculture, USDA-ARS, Leetown, WV 25430, USA

## Abstract

**Background:**

Uncoupling protein 2 (UCP2) belongs to the superfamily of mitochondrial anion carriers that dissociate the respiratory chain from ATP synthesis. It has been determined that UCP2 plays a role in several physiological processes such as energy expenditure, body weight control and fatty acid metabolism in several vertebrate species. We report the first characterization of *UCP2*s in rainbow trout (*Oncorhynchus mykiss*).

**Results:**

Two *UCP2 *genes were identified in the rainbow trout genome, *UCP2A *and *UCP2B*. These genes are 93% similar in their predicted amino acid sequences and display the same genomic structure as other vertebrates (8 exons and 7 introns) spanning 4.2 kb and 3.2 kb, respectively. *UCP2A *and *UCP2B *were widely expressed in all tissues of the study with a predominant level in macrophage-rich tissues and reproductive organs. In fry muscle we observed an increase in *UCP2B *expression in response to fasting and a decrease after refeeding in agreement with previous studies in human, mouse, rat, and marsupials. The converse expression pattern was observed for *UCP2A *mRNA which decreased during fasting, suggesting different metabolic roles for UCP2A and UCP2B in rainbow trout muscle. Phylogenetic analysis including other genes from the UCP core family located rainbow trout UCP2A and UCP2B with their orthologs and suggested an early divergence of vertebrate UCPs from a common ancestor gene.

**Conclusion:**

We characterized two *UCP2 *genes in rainbow trout with similar genomic structures, amino acid sequences and distribution profiles. These genes appeared to be differentially regulated in response to fasting and refeeding in fry muscle. The genomic organization and phylogeny analysis support the hypothesis of a common ancestry between the vertebrate UCPs.

## Background

In living cells, most energy is produced in the mitochondria through oxidative phosphorylation. In this process, the electron flow from reduced substrates to oxygen generates an electrochemical proton gradient across the inner membrane. This force drives the proton back into the matrix and results in ATP synthesis from ADP and P_i_. Uncoupling proteins (UCPs), which are members of the superfamily of mitochondrial anion-carrier proteins, are capable of dissipating the proton gradient across the inner mitochondrial membrane to generate heat while reducing the efficiency of ATP synthesis [1]. The archetypical UCP1 is expressed in brown adipose tissue of mammals and is involved in non-shivering thermogenesis [2,3]. *UCP1 *mRNA has been recently found in ectothermic organisms such as carp, zebrafish and pufferfish [4]. Homologues of UCP1 (UCP2, 3, 4, 5) have been identified from various tissues in vertebrates [5-7] and plants [1,8].

UCP2 has been described in previous studies to play a role in various physiological processes such as body weight control [9-12], fatty acid metabolism [13,14], control of reactive oxygen species [15,16], and negative regulation of insulin secretion [17,18]. No clear thermogenic function has been identified for *UCP2 *[19] but increases of muscle *UCP2 *mRNA in response to fasting has been reported in rat [20,21], human [22] and marsupials [23]. UCP2 appeared along with UCP3 to affect energy partitioning, feed efficiency, body mass index and obesity [6,24].

The present study was designed to characterize *UCP2 *genes in the rainbow trout and investigate their potential as candidate genes affecting traits associated with energy balance and nutrition. To this end, we analyzed the genomic structure, phylogenetic relationships with other UCPs, tissue distribution and expression in muscle of *UCP2 *in response to fasting.

## Results and discussion

### Analysis of cDNA and amino acid sequences

We identified two similar tentative consensus sequences (TC78216 and TC78217) by homology search for *UCP2 *in the TIGR rainbow trout gene index (RTGI). Both TC78216 and TC78217 were found to contain full-length coding sequences from clones tcad0009a.o21 and tcad0008a.b11, respectively. An additional cDNA clone (1RT84B23) containing EST CA344639 which is assigned to TC78216 was also picked, purified and sequenced. Full sequences of tcad0009a.o21, RT84B23 and tcad0008a.b11 were deposited to GenBank and assigned accession numbers [GenBank: DQ295326, DQ295327 and DQ295328].

Similarity analyses between cDNA sequences revealed that the 1,612-bp 1RT84B23 was 90% identical with 1,455-bp tcad0009a.o21. An 157-bp insert in 1RT84B23 accounted for the 10% difference between both sequences. The cDNA clone tcad0008a.b11 was 1418 bp and was 78% and 88% similar to 1RT84B23 and tcad0009a.o21, respectively. For ease of identification, tcad0009a.o21 and tcad0008a.b11 were dubbed *UCP2A *and *UCP2B*, respectively.

**Figure 1:**
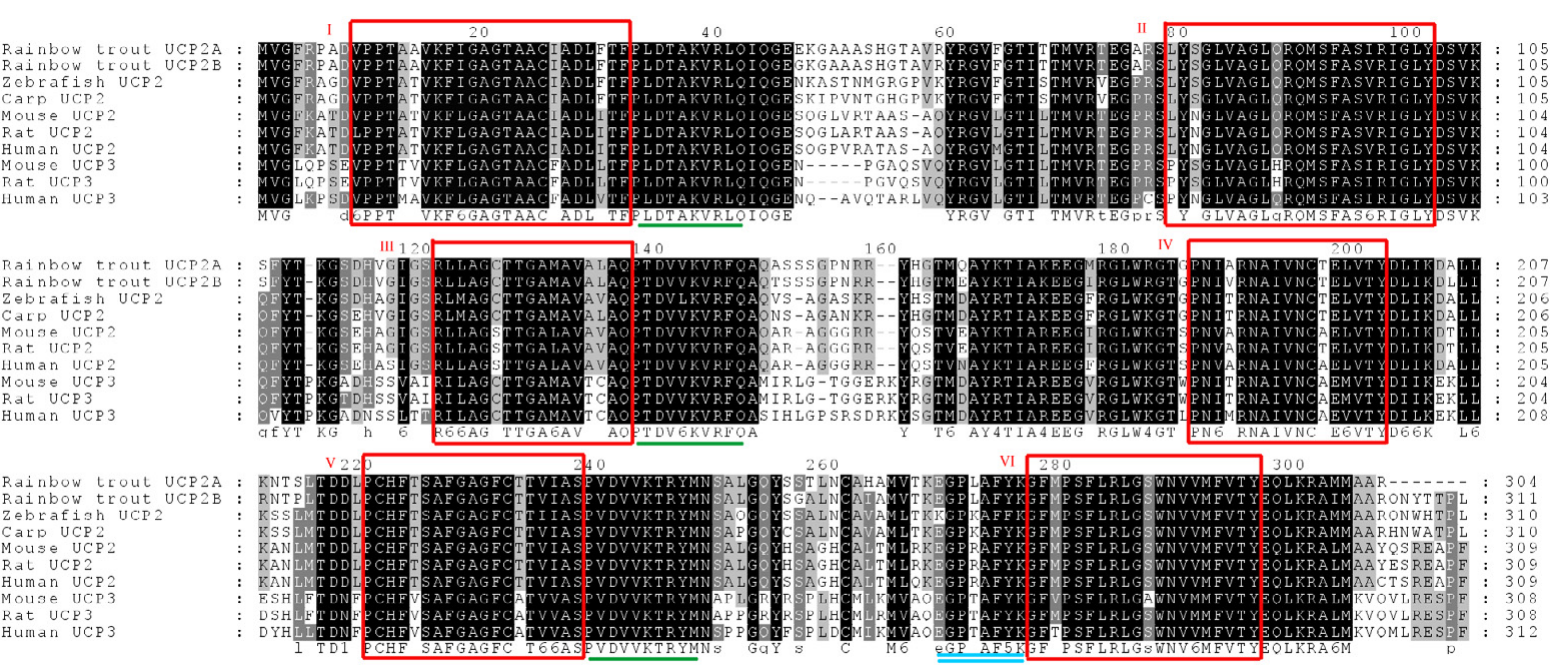
**Multiple amino acid sequence alignment of *UCP2*s**. Sequences include human [Genbank: NM_003355], mouse [GenBank: NM_011671], zebrafish [GenBank: NM_131176], rat [GenBank: AB005143], carp [GenBank: AJ243486] and rainbow trout [GenBank: DQ295326, DQ295328] and UCP3s from human [GenBank: AAC18822], mouse [GenBank: NP_033490] and rat [GenBank: AB006614] obtained using the ClustalW sequence alignment program and illustrated with Genedoc 2.6 [68]. Sequences are presented in single letter code. Gaps, illustrated with a dash, are introduced into sequences to optimize alignment score. Most conserved amino acids are highlighted in black, less conserved ones being in decreasing gray background. Potential transmembrane conserved α-helices domains are boxed and numbered in roman numerals. Proton carrier signatures are underlined once and the potential purine binding domain is underlined twice.

The deduced amino acid sequences of *UCP2A *and *UCP2B *consist of 304 and 311 amino acid residues, respectively (Figure 1). The peptide sequence deduced from 1RT84B23 is a truncated form of that obtained from tcad0009a.o21. The deduced protein, which consisted only of transmembrane domain I and one proton carrier signature, most likely lacks the proton dissipation function as it has been demonstrated that the second transmembrane domain of UCP genes is essential for the anion channel formation [25]. This sequence was therefore discarded from further analysis.

We found 93% similarity between the UCP2A and UCP2B peptide sequences with both containing six transmembrane domains and three proton carrier signatures which define the general triplicate structure of mitochondrial uncoupling proteins [26]. The purine-binding domain involved in the control of coupling efficiency was also identified. Over the protein conserved domains rainbow trout UCP2A and UCP2B diverged only by one amino-acid at position 189 in transmembrane domain IV. The switch from alanine (UCP2A) to valine (UCP2B) should not make any difference in the three-dimensional structure of the peptides as these are both hydrophobic amino acids. On average, both rainbow trout UCP2s showed 83% amino acid similarity with zebrafish UCP2 and 78% amino acid similarity with human, rat and mouse UCP2s. UCP2 and UCP3 have been described to be fairly similar in their peptide sequences and their physiological functions [23,27,28]. Our results showed 69% amino acid similarity between rainbow trout UCP2s and mammalian UCP3s.

The identification of two copies of *UCP2 *genes in rainbow trout genome is consistent with the ancestral whole genome duplication [29,30] and re-diploidization of salmonidae species. Several genes and microsatellite loci in rainbow trout have been previously described to be present in multiple copies [31,32] and Palti *et al*. estimated that approximately two-thirds of the rainbow trout genes remained duplicated [33].

### Phylogenetic relationships

**Figure 2:**
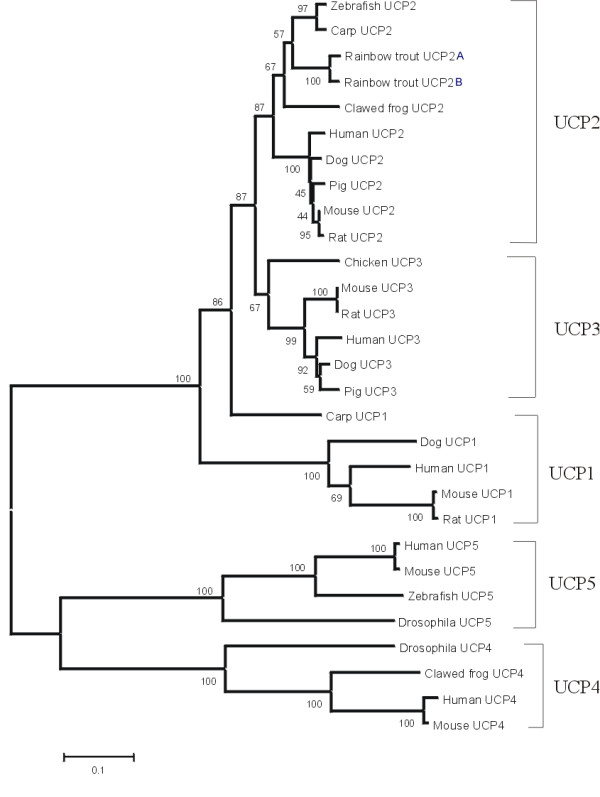
**Evolutionary tree of UCP gene family inferred using the neighbor-joining algorithm**. Genetic distance proportional to branch length was calculated based on amino acid difference (p-distance) with complete deletion of gaps. Bootstrap values computed after 1,000 random replications are indicated at tree nodes. Accession numbers for UCPs are: [GenBank: AAH69556, NM_003355, AAC18822, AF110532, AF078544] for human (*Homo sapiens*) UCP1, *UCP2*, UCP3, UCP4 and UCP5, respectively; [GenBank: NM_009463, AAD17198, NM_009464, NM_028711, NM_011398] for mouse (*Mus musculus*) UCP1, *UCP2*, UCP3, UCP4 and UCP5, respectively; [GenBank: NM_012682, AB005143, AB006614] for rat (*Rattus norvegicus*) UCP1, *UCP2 *and UCP3, respectively; [GenBank: NM_001003046, AB020887, NM_001003047] for Dog (*Canis familiaris*) UCP1, *UCP2 *and UCP3, respectively; [GenBank: NM_131176, BI474135] for Zebrafish (*Danio rerio*) *UCP2 *and UCP5, respectively; [GenBank: AAS10175, AJ243486] for Carp (*Cyprinus carpio*) UCP1 and *UCP2*, respectively; [GenBank: NM_214049, AAD08811] for pig (*Sus scrofa*) *UCP2 *and UCP3, respectively; [GenBank NM_133018.2, AAK92857] for Drosophila UCP4 and UCP5, respectively; and [GenBank: AAH44682, NM_133018] for frog (*Xenopus laevis*) *UCP2 *and UCP4, respectively.

We performed a phylogenetic analysis to classify rainbow trout UCP2A and UCP2B and determine their relationships to UCPs from other species (Figure 2). The tree generated included 27 peptide sequences from the UCP gene family retrieved from public databases. Trees with similar topology to that of Figure 2 were drawn when either Minimum Evolution or Unweighted Pair Group algorithms were performed. Both rainbow trout UCP2 duplicates were grouped together with their UCP2 orthologs in the same clade. Fish (D. rerio, C. carpio and O. mykiss) and frog UCP2 peptides were assigned to a specific cluster, with a high bootstrap (87%), as opposed to mammalian UCP2 genes. Our data showed a close relationship between UCP2 and UCP3 in agreement with previous expression patterns and amino acid sequence similarity suggesting that UCP2 and UCP3 are paralogs [23,27,28]. Moreover both genes have been mapped on the same chromosome in endothermic vertebrates such as human, bovine, porcine and mouse [34-37] and more recently the same evidence was found in ectothermic vertebrates like zebrafish. [4].

Overall, UCP genes were clustered in three major branches at 100% bootstrap value leading to three groups consisting of UCP1-3, UCP-4 and UCP-5. We suggest an early divergence of the UCP family gene into three major clades in agreement with the phylogeny described by Sokolova and Sokolov [38]. These authors also demonstrated that this subdivision could be correlated with a functional specialization among the UCP superfamily.

### Genomic organization of rainbow trout *UCP2*

We identified 32 total BAC clones putatively containing an *UCP2 *gene of which 16 where specific to *UCP2A *and 16 were specific to *UCP2B*. After fingerprinting, most of these BAC clones were assembled into three contigs C1, C2 and C3 containing 12, 7 and 2 clones, respectively. C1 consisted of *UCP2A*-containing BACs while C2 and C3 consisted of *UCP2B*-containing BACs. Two representative BACs were selected from each contig for direct sequencing. Two BAC clones, 243P06 and 398G03, were selected from C1 and C3 for further analysis based on their sequence similarities with cDNA clones tcad0009a.o21 (*UCP2A*) and tcad0008a.b11 (*UCP2B*), respectively. Genomic sequences for *UCP2A *and *UCP2B *genomic sequences have been deposited at NCBI [GenBank: DQ295324, DQ295325].

**Figure 3:**
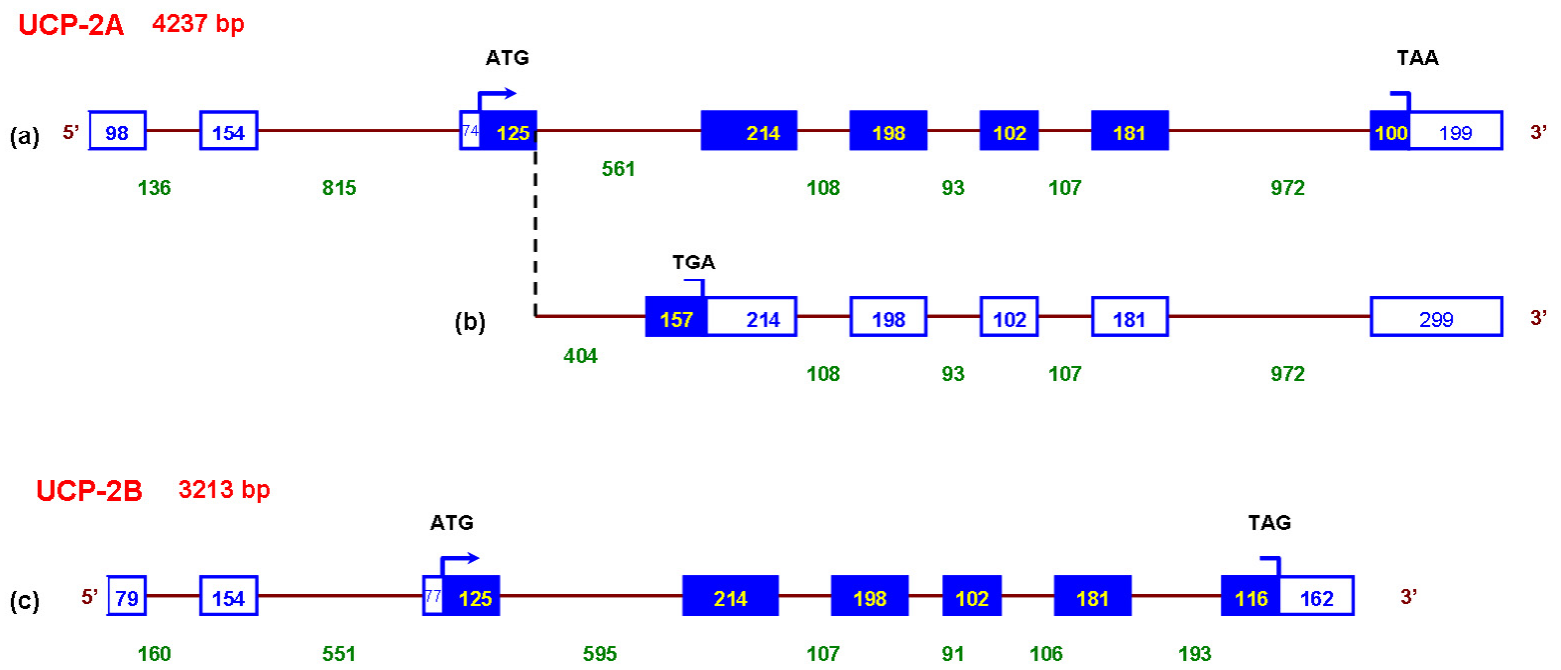
**Schematic representation of the exon-intronstructure of *UCP2A *(a) *UCP2A *splice variant (b) and *UCP2B *(c)**. Lines represent introns, open boxes indicate untranslated exons and shaded boxes represent coding exons. Exon and intron sizes are indicated in base pairs. Exons and introns are not drawn to scale. ATG initiation codon and stop codons are represented by an arrow and a bar, respectively.

Alignment between cDNA and BAC genomic sequences revealed the exon-intron structures of *UCP2A *and *UCP2B *(Figure 3). In each alignment a TATA box was identified on genomic DNA 47 and 45 bp upstream of the 5'end of *UCP2A *and *UCP2B*, respectively. *UCP2A *and *UCP2B *span 4.2 kb and 3.2 kb, respectively. Both rainbow trout *UCP2*s contain 8 exons and 7 introns. Coding sequences for both genes covered 6 exons as described for mammalian *UCP1 *[39], *UCP2 *[40,41] and *UCP3 *[42] and each exon encodes one transmembrane domain of the protein. The analysis of genomic structure showed that 1RT84B23 is a splice variant of tcad0009a.o21. Exon 4 was 157-bp longer and intron 3 was subsequently 157-bp shorter in 1RT84B23 than in tcad0009a.o21 (Figure 3). Exon 4 of 1RT84B23 is not fully translated as a stop codon is encountered in the first 15 nucleotides. In human where *UCP2 *has been well characterized, SNP and insertion/deletion variants of the gene were identified [43-45] and no alternative splice variants have been reported to date.

Similarity between *UCP2A *and *UCP2B *was low in the introns with 60% similarity on average while the average similarity in the exon sequences was 86%. The number and the size of the exons and introns of rainbow trout, human, mouse and zebrafish *UCP2*s are represented in Table 2. The number of exons was identical in these vertebrate organisms although exon lengths were observed to be somewhat variable. Translated exons 4, 5, 6 and 7 were the most conserved in size between species and within rainbow trout. Exon 7 had the same size in all species. These translated exons showed 93% average sequence similarity between rainbow trout UCP2A and UCP2B while untranslated exons were 79% identical. Mutations apparently occur at a slower rate in the coding region than in the untranslated region of duplicated genes. These results show conservation of the structure of *UCP2 *genes coding exons within vertebrates confirming the hypothesis of a common ancestry for *UCP *genes.

**Table 1:** Specific primer sequences for Real-time PCR and corresponding ESTs accession numbers.

Gene	cDNA clones	EST Accession no.	Forward primer 5'→3'	Reverse primer 5'→3'
*UCP2A*	tcad0009a.o21	[GenBank: BX077844, BX077845]	TCCGGCTACAGATCCAGG	CTCTCCACAGACCACGCA
*UCP2B*	tcad0008a.b11	[GenBank: BX079833, BX079834]	TGTAATCACGAGGCATCA	GGATTCTCTAAAGGCGTC
EF1-α	-	[GenBank: AF498320.1]	GGGCAAGGGCTCTTTCAAGT	CGCAATCAGCCTGAGAGGT

**Table 2:** Size in base pairs of *UCP2 *gene exons and introns. Species include rainbow trout [GenBank: DQ295324, DQ295325], human [GenBank: NC_000011.8], mouse [GenBank: NT_039433] and zebrafish [GenBank: NC_007116.1]

No	Rainbow trout *UCP2A*		Rainbow trout *UCP2B*		Human		Mouse		Zebrafish		Exon average length (± SD)	Intron average length (± SD)
	Exon	Intron	Exon	Intron	Exon	Intron	Exon	Intron	Exon	Intron		
1	98	136	79	160	125	1088	103	747	101	125	101.2 ± 16.4	451.2 ± 443
2	154	815	154	551	156	2998	162	2350	145	2554	154.2 ± 6.1	1853.6 ± 1098
3	199	561	202	595	225	156	223	146	221	2701	214 ± 12.4	831.8 ± 1067
4	214	108	214	107	211	868	211	772	214	78	212.8 ± 1.6	386.6 ± 397
5	198	93	198	91	196	80	196	75	195	1051	196.6 ± 1.3	278 ± 432
6	102	107	102	106	101	969	102	286	102	80	101.8 ± 0.5	309.6 ± 378
7	181	972	181	193	181	369	181	319	181	2710	181 ± 0.0	912.6 ± 1049
8	299	-	278	-	451	-	2787	-	286	-	820.2 ± 1101.8	-
Gene size (bp)	4,237		3,211		8,174		8,660		10,774			

We examined introns 1 and 2, untranslated exons 1, 2, untranslated part of exon 3 and approximately 300 bp of genomic sequence upstream of each gene's transcription initiation site as the putative promoter regions. This represented 1.6 and 1.3 kb-long sequence upstream of the translation-initiation site (ATG) of *UCP2A *and *UCP2B*, respectively. Similarity was 59% between overlaps suggesting differentiation between regulatory regions of *UCP2A *and *UCP2B*. The promoter regions of rainbow trout *UCP2A *and *UCP2B*, and the upstream non-coding sequences identified in human (4.5 kb), mouse (3.5 kb) and zebrafish (3.0 kb) *UCP2 *were searched for regulatory element binding sites using the TFSearch engine version 1.3. The results for ten transcription factors frequently identified are summarized in Tables 3 and 4. The identification of regulatory conserved motifs upstream of the five genes is consistent with the phylogenetic relationships previously described between the *UCP2 *genes in human, mouse, zebrafish and rainbow trout. The binding sites of ADR1, CAP, CdxA and HSF were identified in upstream region of all five genes although at different frequency. AML1a (acute myeloid leukemia), MZF (Myeloid zinc finger protein 42) USF (Upstream Stimulatory Transcription Factor) binding sites were identified in mouse and human sequences and absent in fish (rainbow trout, zebrafish). Among the fish *UCP2 *genes, the rainbow trout *UCP2A *regulatory region appeared to be the most unique with almost half of the binding motifs identified in *UCP2B *and zebrafish *UCP2*. Among the regulatory motifs we identified for *UCP2 *genes, USF and CREB have been shown to regulate the expression of *UCP2 *and *UCP1*, respectively [47,48]. Our results suggest, in agreement with previous data in the literature [49,50], that some transcription factor binding sites are conserved across species while others are not.

**Table 3:** Computational identification of putative transcription factor binding sites. Species include rainbow trout (*UCP2A Om*, *UCP2B Om*) zebrafish (*UCP2 Dr*), mouse (*UCP2 Mm*) and human *UCP2 *(*UCP2 Hs*) promoter regions using TFSearch engine version 1.3. Number of representations of each transcription factor binding sites are indicated and "-"indicates less than 2 binding sites.

Genes	ADR1	AML-1a	CAP	CdxA	CREB	HSF	MZF1	Nkx2	SRY	USF
*UCP2A Om*	2	-	2	9	-	5	-	-	-	-
*UCP2B Om*	2	-	2	6	3	9	-	2	4	-
*UCP2 Dr*	4	-	4	18	-	24	-	3	4	-
*UCP2 Mm*	26	2	26	2	-	21	4	2	2	2
*UCP2 Hs*	20	5	20	3	-	19	3	3	5	-

ADR1: Alcohol Dehydrogenase Gene Regulator 1 (*S. cerevisiae*), activator.

AML-1a: Acute Myeloid Leukemia 1; (human); may act as a negative regulator.

Cap: cap signal for transcription initiation signal (vertebrates), activator.

CdxA: chick; Chicken CHox-cad gene for caudal homologue, activator.

CREB: Cyclic-AMP Response Element-Binding Protein, transactivator.

HSF: Heat Shock Transcription Factor (Drosophila), activator.

MZF1: Myeloid Zinc Finger protein MZF1 (human); possibly involved in hemopoietic development.

Nkx2: murine homeobox gene (mouse) modest activator probably important for myocardial development.

SRY: Sex-determining Region Y gene product (human, mouse).

USF: Upstream Stimulatory Transcription, activator (mouse).

**Table 4:** Location of transcription factor binding sites in the promoter regions of rainbow trout *UCP *genes. Positions are indicated relative to the translation-initiation site ATG.

Transcription factors	*UCP2A*	*UCP2B*
ADR1	-707; -533	-1070; -535
CAP	-1045; -40	-1268; -872
CdxA	-1188; -1171; -820; -869; -800; -741; -713; -139; -14	-1281; -1260; -947; -490; -473; -119
CREB	-	-1091; -872; -631
HSF	-1044; -991; -934; -238; -75	-1162; -1112; -896; -783; -731; -672; -605; -449; -78
Nkx2	-	-639; -259
SRY	-	-1253; -1106; -938; -720

### Tissue distribution and response to fasting

**Figure 4:**
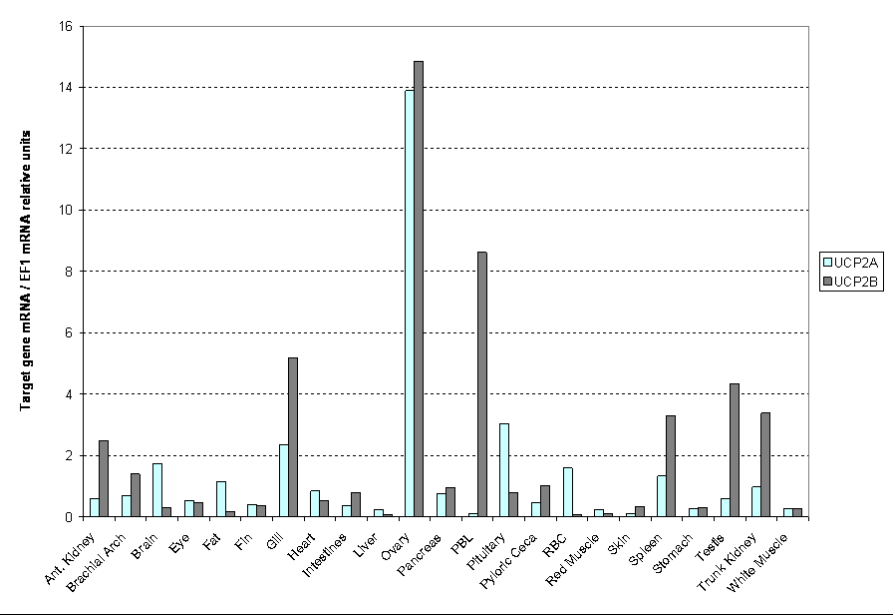
**Tissue distribution of rainbow trout *UCP2A *and *UCP2B *mRNA relative expressions estimated by Real-time RT-PCR**. Various tissues were collected on 2 years old fish weighting approximately 1 kg. Gene mRNA values were normalized to EF1. Means values were computed from 3 technical replicates. Abbreviations: PBL: peripheral blood leukocytes; RBC: Red blood cells.

The distribution of relative expression of rainbow trout *UCP2A *and *UCP2B *was investigated on a panel of 23 tissues from adult fish (Figure 4). Both *UCP2A *and *UCP2B *genes appeared ubiquitously expressed in the body as described in marsupials [23], human [6,46], carp [4] and mouse [7] making the genes likely to influence several physiological processes. The highest relative expression was observed in ovary, peripheral blood leucocytes (PBL), gill, testis, spleen and trunk kidney. In all these tissues except ovary, *UCP2B *mRNA relative amounts were at least twice that of *UCP2A*. In brain, fat, pituitary and red blood cells, *UCP2A *mRNA was more abundant than *UCP2B *mRNA. High amounts of *UCP2 *mRNAs in reproductive organs and in tissues rich in macrophages like PBLs, spleen, and kidney have been reported in several studies. Arsenijevic *et al*. [51] and Kizaki *et al*. [52] suggested a role for UCP2 in immunity or inflammatory responsiveness through the regulation of reactive oxygen species in macrophages. In mouse ovary, *UCP2 *mRNA levels have been described to increase during ovulation as a result of the increasing number of macrophages and neutrophils [53]. In trout, ovaries in maturation are characterized by the presence of many macrophages inside the cytoplasm of the oocytes [54]. The ovaries used in our tissue distribution panel were developing. Therefore, we suggest the high amount of *UCP2 *mRNA in trout ovary could be due to the macrophage-rich developmental stage of the tissue used.

**Figure 5:**
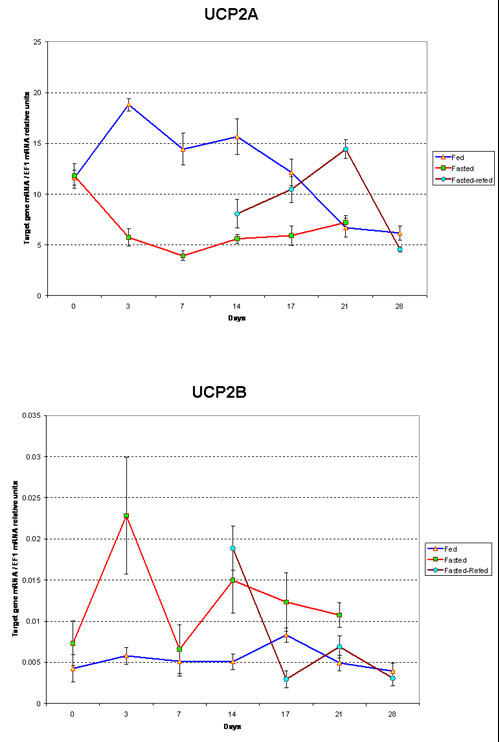
**Rainbow trout *UCP2A *(A) and *UCP2B *(B) mRNA relative expression**. Relative Expression was estimated by Real-time RT-PCR in mixed fiber type muscle of fed and fasted fry at 7 time points after food deprivation and fasted-refed fry at 4 time points on refeeding. Day 0 on chart corresponds to 37 days post hatching for all samples. Data are presented as means ± SEM (n = 4–6)

The initial average weight of the fish used in the current study was 231 mg (225 for the fed group and 236 for the fasted group). Following day 14, the fed fish weighed 475 mg and the fasted fish were 167 mg. At day 21 of fasting, termination of the fasted group, the average weight of the fish was 661 mg, 171 mg and 239 mg for the fed, fasted and refed groups, respectively. Rainbow trout *UCP2A *and *UCP2B *were differentially expressed in response to fasting and refeeding in fry (Figure 5). *UCP2A *mRNA expression was similar between all groups at the beginning of the experiment. Three days after food deprivation, *UCP2A *expression was three times higher in fed fish than in fasted fish. The amount of gene expression remained significantly higher in fed fish than in fasted fish until day 21 of the experiment. Refeeding of fasted animals started at day 14 and resulted in a 2-fold increase of *UCP2A *at day 21. At day 28, refed and fed fish had the same amount of *UCP2A *expression.

At day 0, *UCP2B *expression is similar in all fry. At day 3, expression was 3-fold augmented in fasted fish while no significant increase was found in the fed fish. *UCP2B *expression drastically decreased in fasted fish to the level observed in fed fish at day 7 before a significant increase was observed again at day 14. At days 14 and 21, *UCP2B *was expressed higher in fasted fry than in fed fry. In fed animals, no significant differences were noted between *UCP2B *expression levels throughout the experiment, except at day 17. Refed fish showed the same level of expression of the gene as the fasted fish at day 14. *UCP2B *expression then decreased in refed fish to reach the same level expressed in fed animals at days 21 and 28. The expression pattern of *UCP2B *in response to fasting is consistent with what has been described for other species in the literature. The food deprivation induced-increase in mRNA expression is one of the most spectacular features of UCP2 and UCP3 in rodent and human [20-22,55-58]. The hypothesis is that during fasting when glucose is limiting, upregulation of muscle UCP2 is associated with the shift in muscle substrate utilization primarily towards lipid. On refeeding with a low-fat diet, UCP2 is down regulated allowing the re-use of glucose as major substrate. It has been also demonstrated that a high-fat diet induces up-regulation of UCP2 and UCP3 thus promoting fat utilization over storage [55,57]. The actual mechanism of UCP involvement in fatty acid metabolism is not yet fully understood, but Samec *et al*. [56] established a close relationship between expression of UCP2 and UCP3 and well-known key regulators of lipid oxidation. Our study, unlike most of experiments in the literature, monitored *UCP2 *mRNA levels for a relatively long fasting time (four weeks). Our results suggest that increase of *UCP2B *mRNA is maintained during the whole fasting time, although the higher expression is detected early.

The overall amount of expression of *UCP2B *genes in fry muscle were much lower than what we observed for *UCP2A*. Five times more cDNA was included in Real-time PCR for *UCP2B *primers than was used for *UCP2A *to detect readable amplification signals. As a result, the highest detected amount of *UCP2B *gene *EF1*-normalized expression is 0.023 compared with that of *UCP2A *which was 18.81. In adult fish, as described above *UCP2B *mRNA expression was much higher relatively to *UCP2A *mRNA in some of the tissues. In light of the expression data in response to fasting, *UCP2B *is likely the homolog of the *UCP2 *described in other vertebrates. *UCP2A *is a duplicate form of the gene that has undergone a different evolutionary process leading to a different expression pattern. This is illustrated by the sequence divergence and different cis-regulatory elements identified in the promoter regions of both genes. Further functional characterization of the promoter region and identification of regulatory motifs could help address the differences between mRNA expression of *UCP2A *and *UCP2B*.

## Conclusion

We have identified two *UCP2 *genes in rainbow trout. Both genes were highly similar at the amino acid level and widely expressed in the body. Analyses of the genomic organization and phylogeny data based on protein sequence similarity suggest a close relationship between members of the UCP superfamily. In rainbow trout fry *UCP2A *and *UCP2B *appeared downregulated and upregulated on fasting, respectively. Further characterization of both genes in different physiological and dietary conditions, and developmental stages will assist in better testing and understanding hypothesis related to their specific functions and differentiation.

## Methods

### Search for rainbow trout *UCP2 *cDNA

*UCP2 *sequences from human, mouse and zebrafish *UCP2 *mRNAs [GenBank: NM_003355.2, NM_011671.2, NM_131176.1] were T-BLASTed against the Rainbow Trout Gene Index [59] to identify putative orthologues from rainbow trout [60].

#### Identification and fingerprinting of UCP2 gene-containing BAC clones

Gene specific primers were designed from putative rainbow trout *UCP2 *cDNAs identified by homology searches for screening of the NCCCWA Swanson 10× bacterial artificial chromosome (BAC) library [26] PCR pools according to the manufacturer's directions (Amplicon Express, Pullman, WA). Two sets of primers were designed to screen the BAC library, forward 5'-CACCATGGTGCGTACAGAG-3' and reverse 5'-TTGGGATGGTCTTGTAGG-3'; and forward 5'-TTTATCGGTGCTGGAACAGC-3' and reverse 5'-TAAGAAGCGCGTCCTTGATG-3'. Primer specificities were checked by alignment between cDNAs and by BLAST against NCBI databases.

BACs identified as positive for *UCP2 *genes were fingerprinted using HindIII to identify sets of overlapping clones as previously described [33]. BAC DNA was obtained using the Qiagen Large Construct Kit protocol. Following digestion and electrophoresis, gel images were captured using a Molecular Dynamics Typhoon 9210 Variable Mode Imager and exported as TIF files. Banding patterns were analyzed using Image 3.10b and FPC (Fingerprinted Contigs) V6 software [61] to assemble overlapping BACs into contigs [62].

### Sequence analysis

DNA sequencing was performed using the Big Dye^® ^Terminator Cycle Sequencing Kit and an ABI3100 automatic sequencer (Applied Biosystems, Foster City, CA). cDNA and genomic sequences were obtained by primer walking. Sequences were analyzed using the Sequencher 4.1.4 software (Gene Codes Corporation, Ann Arbor, MI, USA). Exon-intron boundaries were identified by locating 5' donor GT and 3' acceptor AG consensus sequences in a cDNA-genomic DNA gap alignment.

Genomic sequence upstream of the rainbow trout *UCP2 *gene was obtained in order to perform a computational prediction of cis-acting transcription factor binding sites using the TFSearch engine [63] that uses the TRANSFAC databases. Also for each human, mouse and zebrafish, we downloaded the genomic and mRNA sequences from GenBank and aligned them to retrieve the gene structure and locate the translation-initiation site. The upstream sequences including 300 bp upstream on the genomic sequence were then selected for the prediction of transcription factors binding sites. The identifiers of human, mouse and zebrafish *UCP2 *genomic sequence obtained are [GenBank: NC_000011.8, NT_039433, NC_007116.1], respectively. Sequence obtained for promoter analysis was located on human chromosome 11q13 (73,363,064-73,367,806), on mouse chromosome region 7E2 (18,239,174-18,242,911) and zebrafish chromosome 5 (60,833,577-60,836,877).

### Experiment design and tissue collection

Experiments were conducted on rainbow trout fry hatched at the National Center for Cool and Cold Water Aquaculture (Leetown, WV). Thirty three fish at 32 days post-hatching (dph) were placed into each of 18 × 2 4L buckets. The buckets were supplied with flow-through oxygen saturated well water at 12.5 + 0.2°C, and a photoperiod of 12 h light -12 h dark. Fish were fed a commercial diet, Finfish Starter Meal, (57% protein and 15% fat; Ziegler Bros. Inc., Gardner PA) at 2% body weight daily. Fish were divided into three treatment groups: 1) fed fish as controls, 2) fasted fish, and 3) fish fasted the first 14 days of the experiment and re-fed thereafter. The experiment was initiated 37 dph, shortly after the completion of yolk-sac absorption. Muscle tissues were collected on days 0, 3, 7, 14, 17, 21 and 28 of the experiment. Each tank was only sampled once and fish from two tanks for each treatment were sampled. At sampling, fish were anesthetized with 200 mg/L Tricaine and mixed fiber type muscle pools from four individuals were excised and immediately frozen in liquid nitrogen then stored at -80°C until RNA extraction. All animal handling and sampling procedures were reviewed and approved by the NCCCWA Institutional Animal Care and Use Committee.

Tissues were collected from branchial arch, brain, eye, fat, gill, heart, intestines, liver, ovary, pancreas, peripheral blood leukocytes, red and white muscles, pituitary, pyloric ceca, red blood cells, skin, spleen, stomach and testis on healthy 2-year old adult fish (approximately 1 kg in mass) for the survey of *UCP2 *mRNA expression in the rainbow trout body.

### RNA isolation and first strand cDNA synthesis

Total RNA was extracted using Tri-reagent (Sigma, St Louis, Missouri) according to the manufacturer's protocol. Total RNA was precipitated in ethanol, washed and dissolved in nuclease-free water. RNA was treated with DNAse to eliminate genomic DNA contamination and re-extracted using Tri-reagent. RNA concentrations were estimated by spectrophotometry and integrity was checked on agarose gels. First strand cDNA synthesis was performed in a 40 μl reaction volume containing 2 μg total RNA, 1 μg of Random Hexamer primers, 1× M-MLV reaction buffer, 500 μM of dNTPs, 200 units of M-MLV reverse transcriptase (Promega, Madison, USA) and 25 units Recombinant Rnasin^® ^Ribonuclease Inhibitor (Promega, Madison, USA). The mix was incubated at 37°C for 60 min with a final denaturation at 95°C for 5 min. Reactions were stored at -20°C until further use.

### Real-time PCR

Specific forward and reverse primers were designed for each target and reference gene cDNA (Table 1) on the boundary of two consecutive exons or in intron flanking regions to prevent amplification of contaminating genomic DNA or to make it detectable on a gel. Either way, amplicons were run on gel to confirm their uniqueness and size expectation prior to real-time PCR runs. Real-time PCR was performed in a 15 μl reaction volume containing 1 μl of cDNA template, 250 nM of each forward and reverse primers and 7.5 μl of SYBR Green PCR Master Mix (Applied Biosystems, Foster City, USA). Amplifications were performed on the ABI PRISM 7900 Sequence Detection System real-time cycler using the following program: 50°C for 10 min, 95°C for 15 s, 40 cycles of 95°C for 15s, 60°C for 30 s, 72°C for 30s and a dissociation stage at 95°C for 15s, 60°C for 15 s and 95°C for 15s. Dissociation curves were analyzed to detect non-specific amplification. Non-template controls were included in each run and checked for no amplification. The amount of transcript in each sample was quantified using the standard curve method [65]. The standard curve was constructed using 10 dilutions (1 to 1/1000) of a pool of cDNA. Gene expression in tissues was rendered as a ratio of target gene versus rainbow trout *Elongation Factor-1α *(*EF1-α*, GenBank Accession Number AF498320.1) as reference gene. We surveyed *EF1-α*, *β-actin*, *TATA box Binding protein a *(*TBP-a*) and *18S rRNA *as potential internal control genes for RT-PCR (data not shown). Of the housekeeping genes tested, *EF1-α *demonstrated the most consistent Ct values between treatments (average 23.7 ± 1.1). *EF1-α *has been previously demonstrated to be a reliable housekeeper in several tissues of Atlantic salmon [64].

The real time PCR mRNA expression data were analyzed using the Statistica software version 6 (StatSoft. Inc., Tulsa, OK, USA). Differences in *UCP2 *mRNA expression between treatments were assessed at each time point using the Kruskal-Wallis non-parametric test. A pair wise post hoc comparison of means was performed when difference between treatments was significant at the 0.05 threshold.

### Phylogenetic inference

Twenty seven UCP peptide sequences were identified and retrieved from public databases using keywords and BLAST searches. Multiple sequence alignments were conducted using Clustal W [66]. Sequence alignments were used as an input file to construct a consensus phylogenetic tree by the neighbor joining method using MEGA version 3 [67]. Reliability of tree was assessed by bootstrapping using 1,000 random replications.

## Authors' contributions

IC carried out the molecular genetics studies, sequencing, sequence alignment, statistical analysis, and drafted the manuscript. SAG participated in the gene identification process, designed the experiment and extracted the RNA. YP contributed to the BAC fingerprinting. JY and CER participated in the study design and coordination. All authors read and approved the final manuscript.
